# Evolutionary diversification of cryophilic *Grylloblatta *species (Grylloblattodea: Grylloblattidae) in alpine habitats of California

**DOI:** 10.1186/1471-2148-10-163

**Published:** 2010-06-02

**Authors:** Sean D Schoville, George K Roderick

**Affiliations:** 1 Department of Environmental Science, Policy and Management, University of California, Berkeley, 137 Mulford Hall #3114, Berkeley, CA 94720-3114, USA

## Abstract

**Background:**

Climate in alpine habitats has undergone extreme variation during Pliocene and Pleistocene epochs, resulting in repeated expansion and contraction of alpine glaciers. Many cold-adapted alpine species have responded to these climatic changes with long-distance range shifts. These species typically exhibit shallow genetic differentiation over a large geographical area. In contrast, poorly dispersing organisms often form species complexes within mountain ranges, such as the California endemic ice-crawlers (Grylloblattodea: Grylloblattidae: *Grylloblatta*). The diversification pattern of poorly dispersing species might provide more information on the localized effects of historical climate change, the importance of particular climatic events, as well as the history of dispersal. Here we use multi-locus genetic data to examine the phylogenetic relationships and geographic pattern of diversification in California *Grylloblatta*.

**Results:**

Our analysis reveals a pattern of deep genetic subdivision among geographically isolated populations of *Grylloblatta *in California. Alpine populations diverged from low elevation populations and subsequently diversified. Using a Bayesian relaxed clock model and both uncalibrated and calibrated measurements of time to most recent common ancestor, we reconstruct the temporal diversification of alpine *Grylloblatta *populations. Based on calibrated relaxed clock estimates, evolutionary diversification of *Grylloblatta *occurred during the Pliocene-Pleistocene epochs, with an initial dispersal into California during the Pliocene and species diversification in alpine clades during the middle Pleistocene epoch.

**Conclusions:**

*Grylloblatta *species exhibit a high degree of genetic subdivision in California with well defined geographic structure. Distinct glacial refugia can be inferred within the Sierra Nevada, corresponding to major, glaciated drainage basins. Low elevation populations are sister to alpine populations, suggesting alpine populations may track expanding glacial ice sheets and diversify as a result of multiple glacial advances. Based on relaxed-clock molecular dating, the temporal diversification of *Grylloblatta *provides evidence for the role of a climate-driven species pump in alpine species during the Pleistocene epoch.

## Background

In the present climate, many low-latitude mountain ranges are environmentally isolated from one another by intervening low elevation habitat. Geological and paleoecological data indicate that cooling global climate led to the historical growth of alpine glaciers [1,2] and to down-slope range shifts of montane vegetation [3,4]. It has been argued that ice age climates provided environmental corridors between mountain ranges that enabled arctic and alpine populations to migrate into and among sky-islands [5,6]. This hypothesis suggests that the expansion of continental and alpine glaciers during recent climatic cycles of the Pliocene and Pleistocene epochs facilitated spatial expansion of populations in alpine environments and, subsequently, contraction of these glaciers led to the diversification of geographically isolated lineages.

Molecular genetic studies support this hypothesis in several ways. First, several wide-ranging arctic species also inhabit alpine habitats [7] and these isolated populations exhibit recent genetic divergence [8-11]. This is most easily explained by colonization events or gene flow during the last glacial cycle. Second, many alpine species comprise closely related populations separated on geographically distant mountain ranges. These patterns are evident in mountainous regions in North America [12,13], Japan [14,15], Europe [e.g. [16]], and New Zealand [17]. Finally, several molecular studies examining divergence patterns of populations on separate mountains have detected low levels of gene flow during the divergence process, implying connectivity during glacial periods [18,19]. Fossil remains also suggest that winged insects [20-22] and plants commonly exhibit widespread range shifts during glacial cycles [3,23].

Most of the alpine organisms exhibiting these spatial patterns are highly vagile species. In contrast, many alpine species that have low vagility are considered to be restricted endemics and often form species complexes within mountain ranges [24]. The phylogeographic history of low vagility organisms might therefore exhibit different patterns [25,26] and can offer several important contributions to the study of alpine biogeographic history. First, population diversification of poorly dispersing species might provide insight on the effects of alpine glaciations within mountain ranges, in particular by delineating the spatial location of climatic refugia. Second, the temporal pattern of diversification within and between these species might provide information on the relative impact of different glacial cycles because poorly dispersing populations are more likely to diverge genetically due to the stochastic effects of genetic drift [27]. Third, low vagility species provide an opportunity to understand the dispersal history of populations in alpine environments. For example, these species clarify whether shifts in elevation are necessary to the survival of alpine populations, or if populations managed to survive in high elevation refugia [i.e. ice-free nunataks; [28]]. Similarly, under a "progression-rule" biogeographic model [29], ancestral-descendent relationships could be used to infer the direction of colonization into mountain ranges by a dispersing population.

In this study, the evolutionary history of a low vagility species complex is examined within California. *Grylloblatta *species (Grylloblattodea: Grylloblattidae), or ice-crawlers, are members of an ancient lineage that evolved during the radiation of neopterous insects [30]. All *Grylloblatta *species are cryophilic, with an obligate link to stable near-freezing temperatures. Acute physiological temperature tolerance ranges from -8.5 to 10°C in an undescribed species on Mt. Ranier [31], and low temperature tolerance is a conserved trait in the genus [32]. Very few arthropods survive prolonged exposure to temperatures experienced on snow fields, but *Grylloblatta *have a foraging strategy utilizing snow-fields, where wind-blown insects and organic detritus are deposited (into the "Aeolian ecosystem") and form an energy rich resource for scavenging insects [33]. While five extant genera of Grylloblattidae are known, less than thirty species have been described. These species occur in northeastern Asia (Korea, Japan, China and Russia) and western North America (Canada and the western United States). A single genus, *Grylloblatta*, occurs in North America with eleven described species, five of which occur in California. In California, *Grylloblatta *species are restricted to montane areas, caves, and deep canyons, and within these habitats remain in close proximity to permanent ice and deep retreats in rock outcrops. Previous to this study, most of the California species were known from type localities and are extremely rare in entomological collections.

Kamp [34,35], working on populations of grylloblattids in northeastern California and the Canadian Rocky Mountains, proposed that contemporary populations were relicts of populations broadly distributed in periglacial habitat during glacial periods. He hypothesized that 1) populations expanded their geographic range during glacial cycles and that 2) alpine populations colonized high elevation habitat after the retreat of glacial ice. We set out to test Kamp's [34] ideas by studying the genetic relationships of California *Grylloblatta *populations in the context of alpine glacial cycles in California. We attempt to explicitly test whether the diversification of *Grylloblatta *in California is intimately tied to glacial-interglacial fluctuations during the Pleistocene. Previous studies have shown that low vagility organisms often exhibit deep genetic breaks due to stochastic processes [25,26]. While these genetic breaks can occur at random in continuously distributed populations [36], they also occur due to evolutionary and environmental events that impede gene flow [37]. Multi-locus phylogenies offer a way to distinguish between random and non-random genetic breaks [38]. A previous multi-locus phylogeny of grylloblattid populations in both the Pacific Northwest and northeastern Asia [39] showed a recurrent pattern of deep genetic subdivision over a small geographical area, suggesting that California grylloblattids might provide similar levels of phylogenetic substructure. We therefore utilize genetic sequence data from multiple independent loci to infer the population genetic history, phylogenetic relationships and diversification time of California *Grylloblatta *species. Our results support Kamp's hypothesis that *Grylloblatta *populations expanded spatially at the edges of glacial habitat. Furthermore, we provide evidence that Pleistocene climatic shifts have had a direct role in *Grylloblatta *species formation.

## Results

### Population Genetic Patterns of *Grylloblatta*

**Figure 1:**
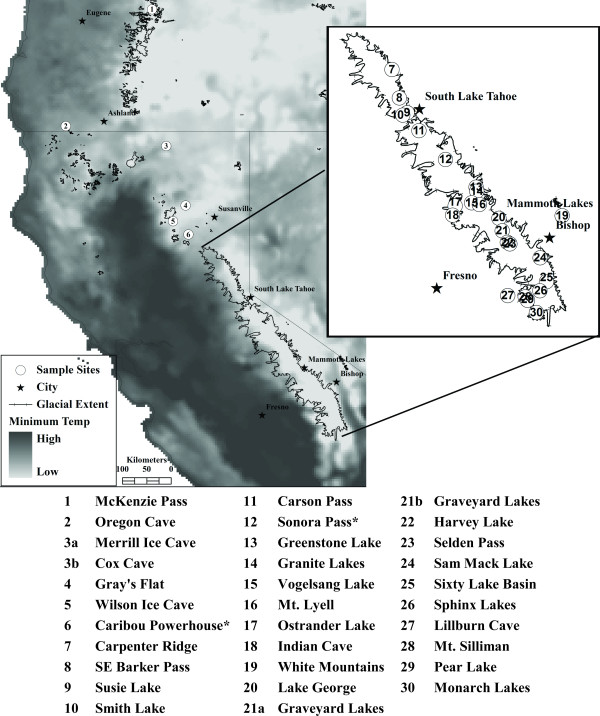
**Sampling locations of *Grylloblatta***. Location of sampled *Grylloblatta *populations in California and Oregon, overlaid on an mean minimum annual temperature surface for 1971-2000 [81] and an outline of glacial ice extent during the last glacial maximum [82]. Type localities of species indicated with an asterisk.

A total of 132 individuals of *Grylloblatta *from 30 sites (Figure 1; Additional file 1 Table 1S) were screened for genetic variation at four genes. There were 52 unique *COII *haplotypes, with one individual (Lillburn Cave) having two highly divergent copies. These copies were amplified with two different primer sets, never co-amplified, and yet translate into appropriate amino acid residues. Although it is unclear whether or not this individual has acquired a nuclear mitochondrial DNA gene (numt), or is heteroplasmic, analyses using alternate copies did not significantly alter phylogenetic results. The copy amplified from the least degenerate mitochondrial primers was chosen for subsequent divergence time analyses. There were 12 unique sequences of the nuclear *histone *(*h3*) gene, 16 unique sequences of the nuclear *18 S *gene, and 18 unique sequences of the nuclear *28 S *gene. The combined sequences included a total of 5,542 sites (with gaps), with considerable rate variation among genes (Table 1). All sequences have been deposited in NCBI's GenBank (Accessions: FJ918575-FJ918687, GU013769-GU013770; Additional file 2 Table 2S).

**Table 1:** Properties of gene partitions used in phylogenetic analysis.

	Number of Basepairs	Variable Site %	Gap %	Relative Mean Rate of Substitution*
COII	742	19.27	0	1.033E^-4^
Histone	327	5.49	0	1.381E^-5^
18S	2,052	3.21	0.11	3.216E^-6^
28S	2,420	5.37	1.89	3.333E^-6^

*Relative substitution rates are based on a fixed mean rate of evolution across lineages (1/time × 10^-4^).

In five well-sampled alpine populations, within-population genetic variation is quite low (Table 2). Mitochondrial DNA variation approaches zero in the Sonora Pass, White Mountain and Graveyard Lakes populations (range *θ_S _*per bp = 0.000-0.001, range *θ_π _*per bp = 0.000-0.001). In contrast, the Barker Pass and Susie Lake populations exhibit slightly higher mtDNA variation (range *θ_S _*per bp = 0.004-0.007, range *θ_π _*per bp = 0.006). In all populations, variation in the nuclear gene *histone *is low and approaches zero. Tests for deviation from neutral equilibrium are non-significant in most populations, although some show a trend towards negative *D *and *F_S _*values. The Susie Lake population exhibits significant negative *D *and *F_S _*values in the *histone *locus, possibly indicating recent population expansion. Mismatch distribution tests for most populations across loci were consistent with a model of sudden population expansion, the exception being the Barker Pass population for *COII*. Under a spatial population expansion model, mismatch distributions were qualitatively similar (results not shown).

**Table 2:** Population genetic summary statistics in five alpine populations of *Grylloblatta*.

	Barker Pass (n = 17)	Susie Lake (n = 22)	Sonora Pass (n = 12)	White Mountains (n = 11)	Graveyard Lakes (n = 12)
	
Cytochrome Oxidase subunit II					
*θ_S_*(per bp)	0.004	0.007	0.001	0.000	0.001
*θ_π_*(per bp)	0.006	0.006	0.001	0.000	0.001
Tajima's *D*	1.73	-0.62	0.02	-1.13	-0.38
Fu's *F_S_*	4.31	-2.21	-0.62	-0.41	-0.36
SSD	0.21*	0.03	0.02	0.02	0.02
Raggedness	0.33	0.07	0.17	0.44	0.20
**Histone**					
*θ_S_*(per bp)	0.001	0.001	0.000	0.001	0.002
*θ_π_*(per bp)	0.001	0.000	0.000	0.001	0.004
Tajima's *D*	-0.19	-1.48*	0	-0.64	1.65
Fu's *F_S_*	0.28	-2.86*	0	-0.18	-1.13
SSD	0.32	0	0	0.02	0
Raggedness	0.37	0.68	0	0.46	0.08

*Statistical tests significant at α = 0.05 are labeled with an asterisk.

### Estimates of Phylogenetic Relationships

**Figure 2:**
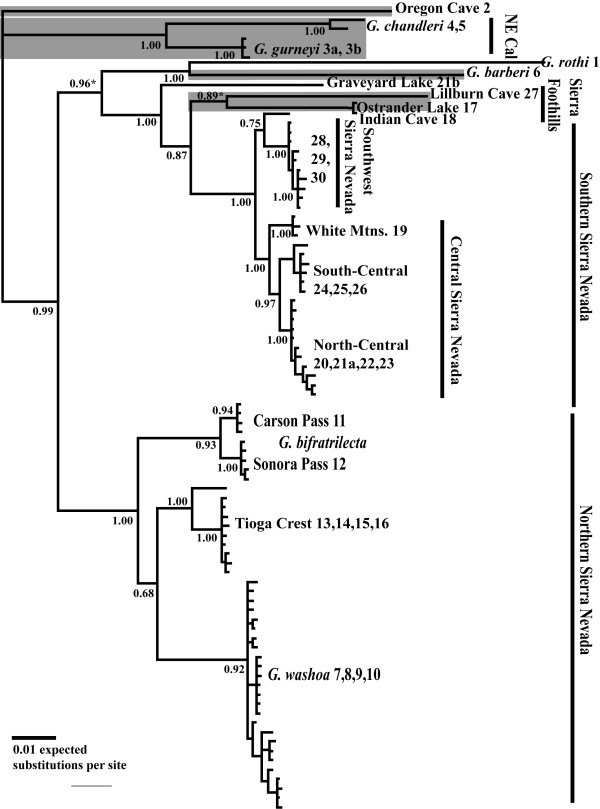
**Bayesian phylogeny of California *Grylloblatta***. Concatenated phylogeny with Bayesian posterior probability support values shown at the nodes. Nodes conflicting with the maximum likelihood topology indicated by asterisks and low elevation populations indicated by shading in gray. Sample site numbers follow names at tips.

**Figure 3:**
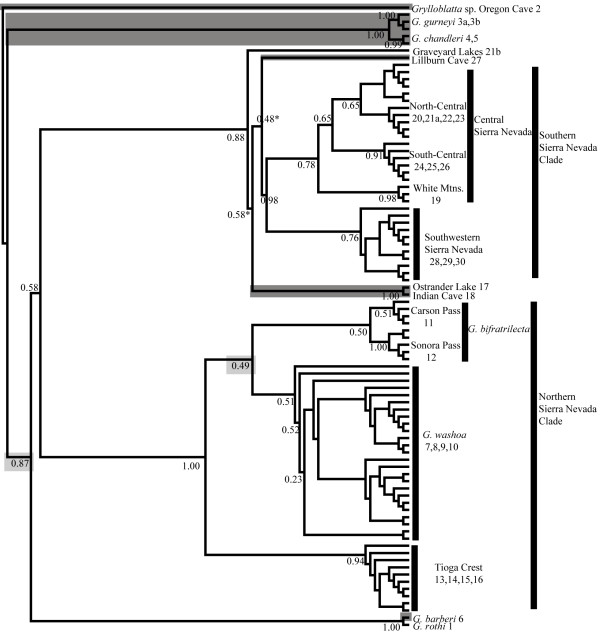
**Maximum likelihood phylogeny of California *Grylloblatta***. Maximum likelihood topology based on 1000 bootstrap replicates. Nodes conflicting with the Bayesian topology indicated by asterisks and low elevation populations indicated by shading in gray. Sample site numbers follow names at tips.

**Figure 4:**
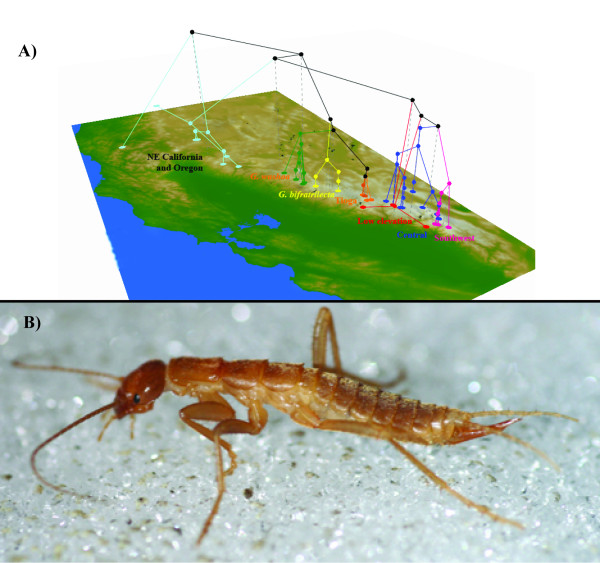
**Geographic distribution of alpine *Grylloblatta *lineages**. A) Geographic distribution of genetically distinct alpine lineages in the Sierra Nevada overlaid on a topographic base map of California [82]. B) Adult female *Grylloblatta *sp. from the White Mountains.

The concatenated phylogenies estimated from the unrooted Bayesian analysis (Figure 2) and the maximum likelihood bootstrap analysis (Figure 3) are very similar. Notable differences in the relationships of a few lineages (see gray highlighting) under these two methods largely concern the order of early branching events in the phylogeny. Separate Bayesian analyses of the mitochondrial data (Additional file 3 Figure 1S) and nuclear data (Additional file 4 Figure 2S) provide similar topologies to the Bayesian concatenated phylogeny, although fine scale geographic structure is only evident in the mitochondrial data. The concatenated phylogenetic analysis identifies three to four major lineages, with substantial phylogeographic structure within these lineages (Figure 4). The first lineage represents the Oregon Cave sample of southern Oregon. The second lineage includes two unique taxa, *G. gurneyi *and *G. chandleri*, sampled from northeastern California. The third lineage includes all individuals from the Sierra Nevada. In the maximum likelihood analysis, a fourth lineage is identified, which is nested within the Sierra Nevada lineage in the Bayesian analysis. This lineage includes *G. rothi *(Three Sisters, Oregon) and *G. barberi *(Feather River, California), which form a sister group in both analyses. Within the Sierra Nevada lineage, there is further subdivision into two major clades, including a northern Sierra Nevada clade and a southern Sierra Nevada clade (9.8% uncorrected mtDNA divergence). Within each of the Sierra Nevada clades there are strongly supported monophyletic groups that are geographically subdivided. The northern Sierra Nevada includes *G. bifratrilecta*, *G. washoa*, and a Tioga Crest subclade (5.6-7.1% uncorrected mtDNA divergence). The southern Sierra Nevada clade includes a southwest Sierra Nevada and a central Sierra Nevada subclade (3.1% uncorrected mtDNA divergence), as well as a deeply divergent subclade including Ostrander Lake-Indian Cave, Lillburn Cave, and a unique individual at Graveyard Lakes. The unusual Graveyard Lakes specimen is a juvenile which was sympatric and actively foraging with grylloblattids from the Central Sierra Nevada lineage, yet exhibits 6.6% uncorrected mtDNA divergence as well as unique nuclear alleles. The subclade including Ostrander Lake-Indian Cave, Lillburn Cave and the Graveyard Lake specimen are sister to the alpine populations in the southern Sierra Nevada. There is further geographic subdivision within the alpine subclades, notably north ("Carson Pass") to south ("Sonora Pass") in *G. bifratrilecta *and northeast ("White Mountains"), north ("North-Central"), and south ("South-Central") in the Central Sierra Nevada subclade.

### Temporal Pattern of Diversification

Time to Most Recent Common Ancestor (*T_MRCA_*) was estimated using a Bayesian relaxed clock model with two different methods: an unscaled, fixed evolutionary rate and a calibrated molecular clock (Table 3). Unscaled estimates of *T_MRCA _*provide a representation of the tempo of diversification, unbiased by a prior on the molecular substitution rate. In the alpine populations, pulses of diversification are evident at several different time intervals, first in the diversification within the northern and southern Sierra Nevada clades, then in the diversification of subclades (*G. bifratrilecta*, *G. washoa*, Tioga Crest, Central Sierra Nevada, and Southwest Sierra Nevada), and finally in the diversification within populations of these subclades (White Mountains, Carson Pass and Sonora Pass). The time of divergence roughly doubles across these different levels and this is clear in the unscaled relaxed clock estimates, as well as the clock analysis. However, median estimates of *T_MRCA _*in both methods result in large confidence intervals and the lineage through time plot (Additional file 5 Figure 3S) does not indicate synchronous pulses of diversification. Instead, divergence rates are shown to be high in the past relative to a constant-rate model, with a subsequent decline and a more recent recovery.

**Table 3:** Estimated Time to Most Recent Common Ancestor under a relaxed clock model.

	Unscaled *T_MRCA_*^1^			COII Calibrated *T_MRCA_*^2^		
	Median	95% Low	95% High	Median	95% Low	95% High
California	696.17	372.43	1177.92	4.53MY	2.59MY	7.45MY

Northeastern California	324.12	146.08	571.70	1.28MY	498,100	2.47MY

G. gurneyi	40.69	3.35	135.96	139,600	9,430	446,100
G. chandleri	110.81	27.18	228.72	239,600	38,680	595,500
						
Division of Sierra Nevada Clades	613.79	349.42	1142.14	3.94MY	2.08MY	6.62MY

Northern Sierra	391.26	198.67	816.92	1.94MY	994,800	3.36MY

G. bifratrilecta	148.10	54.53	299.44	798,500	298,900	1.58MY
Carson Pass	45.84	10.21	126.57	245,600	45,040	625,400
Sonora Pass	62.47	7.45	162.28	195,000	38,240	478,500
G. washoa	189.92	90.52	381.93	727,200	302,400	1.50MY
Tioga Crest	219.01	63.87	459.42	967,300	364,200	1.85MY
						
Southern Sierra	419.23	220.56	707.23	2.52MY	1.32MY	4.28MY

Low Elevation Sierra Populations	417.75	241.96	730.51	2.54MY	1.35MY	4.31MY
Southwest Sierra Nevada	189.90	74.48	350.74	806,100	330,400	1.52MY
Central Sierra Nevada	213.16	108.11	372.05	814,600	380,000	1.45MY
White Mountains	52.10	6.35	145.69	179,800	17,320	505,900

1. Based on a fixed mean rate of evolution across lineages (1/time × 10-4).

2. Based on a mean rate calibration for COII (1.5%/million years).

The calibrated molecular clock estimates have large confidence intervals, but the median estimates of *T_MRCA _*suggest *Grylloblatta *species inhabited California before the onset of the Pleistocene (~4.53 million years B.P., Table 3). *T_MRCA _*of major lineages in the Sierra Nevada are estimated to be in the Early Pleistocene (~1.94 MY for the Northern Sierra Nevada clade, ~2.52 for the Southern Sierra Nevada clade) and during the Early to Middle Pleistocene for geographically isolated subclades in the Sierra Nevada.

## Discussion

### Phylogenetic Relationships among California *Grylloblatta*

Previous phylogenetic analysis [39] demonstrated that North American *Grylloblatta *are monophyletic and derived from a northeast Asian grylloblattid ancestor, and that *Grylloblatta *was split into two deeply divergent lineages occupying the northern Cascades and southern Oregon-California. By increasing the taxonomic and spatial sampling of *Grylloblatta *in California, our analysis reveals a deep split in the Sierra Nevada with a high degree of local geographic subdivision. Both Bayesian and maximum likelihood methods identify a basal polytomy of three major lineages. These lineages include an Oregon Cave lineage, a northeastern California lineage, and a Sierra Nevada lineage. A fourth lineage is identified in the maximum likelihood analysis, comprised of southern Cascades *G. rothi *and northern Californian *G. barberi*, but is alternatively nested in the Sierra Nevada lineage in the Bayesian analysis. This conflict might result from differences in methodological assumptions and/or long-branch effects. All currently recognized species in California form discrete monophyletic groups and correspond to subclades within the major lineages. The northeastern California lineage includes *G. gurneyi *and *G. chandleri*. The northern Sierra Nevada clade includes *G. bifratrilecta *and *G. washoa*. The monophyly of these groups indicates that morphological criteria have been useful in delineating distinct evolutionary lineages. However, other monophyletic groups of similar levels of genetic divergence are present in the Sierra Nevada, including populations along the Tioga Crest, the southwest Sierra, and the central Sierra. Several other specimens from unique low elevation sites (Ostrander Lake and Lillburn Cave) are highly divergent from and sister to alpine subclades. Finally, one specimen collected at Graveyard Lakes is genetically distinct, with unique alleles at all loci (unshared by 12 other individuals at that site). This specimen may represent a sympatric population that has come into contact but is reproductively isolated from the Central Sierra Nevada lineage. Most of the Sierra Nevada populations sampled in our study represent new localities and several monophyletic clades may represent undescribed species.

### Biogeographic Patterns of Lineage Diversification

The diversification of grylloblattids in North America has taken place over a large geographic region that has experienced a complex geological and climatic history [1]. Despite dramatic environmental events, including periodic volcanic eruptions, *Grylloblatta *have managed to persist and recolonize alpine habitats [40]. They are currently found in small, relictual populations where local microenvironments provide cold temperature retreats. The major division of North American *Grylloblatta *occurs between a northern Cascades-Rocky Mountain clade and a California-Southern Oregon clade [39]. This provides little information about the geographic origin of Californian taxa, but indicates that a common ancestor diversified across western North America in the distant past. Furthermore, a basal polytomy in our phylogenetic analysis leaves the relationship of major California lineages unresolved. The Oregon Cave lineage is isolated in the lower slopes of the Siskiyou Mountains, which stretch south into the California Trinity Alps and Mount Shasta region. This population is at the southern end of the Cascades Range and represents the most likely source of colonization into California. However, the unusual relationship and placement of *G. rothi *and *G. barberi *suggests that there may have been multiple dispersal events between Oregon and California, perhaps utilizing other biogeographic corridors.

Relationships of the remaining California taxa are particularly interesting, because these species are a mix of low elevation relict populations and alpine populations. Kamp [34] proposed that taxa in northeastern California were relicts of populations broadly distributed in periglacial habitat during glacial periods. The geographical location of widely separated populations of *G. chandleri *supports this hypothesis, as these populations lie within the periglacial zone of Mt. Lassen. *G. chandleri *and *G. gurneyi *form a highly divergent monophyletic group in northeastern California, suggesting that these low elevation populations have been isolated for a long period of time. Time to Most Recent Common Ancestor, based on fossil-derived substitution rates, suggests diversification within these species (~239,600 years B.P. for *G. chandleri *and ~139,600 for *G. gurneyi*) was initiated during the early stages of glacial cooling phases [41]. Other low elevation populations in our study, including *G. barberi*, Ostrander Lake, and Lillburn Cave, are distinct from and sister to alpine lineages. These taxa do not appear to be recent remnant populations of alpine populations that moved down-slope during glacial periods, but instead seem to share ancestry with populations that gave rise to alpine lineages. All populations in the Sierra Nevada share a single common ancestor, suggesting one dispersal event gave rise to a diverse group of alpine lineages.

Within the alpine lineages, there is a high degree of substructure reflecting the dispersal limitation of populations and, perhaps, the existence of geographical barriers. Some subclades (Central Sierra Nevada, Southwest Sierra Nevada, *G. barberi*, Tioga Crest) have unique mitochondrial haplotypes at all geographical localities sampled, but other well-sampled populations (i.e. *G. washoa*) have shared haplotypes across sites. Current sampling does not clearly elucidate whether nunataks (ice-free high elevation habitat) were important in the persistence of alpine populations and the preservation of genetic variation. It is clear that alpine populations are subdivided north to south, with unique lineages occupying major drainage basins. These drainages were the principal course of alpine glacial growth and it is probable that *Grylloblatta *populations tracked suitable habitat along the periglacial edge environment of these growing glaciers. The White Mountains, lying east of the Sierra Nevada at the edge of the Great Basin, has a population of *Grylloblatta *closely related to Central Sierra Nevada populations. Currently, a distance of roughly 30 miles separates the two ranges in the Long Valley region (elevation >2,125 m). Historically, a high plateau connected these mountain ranges, but subsidence along the Owens Valley fault (beginning 3 million years ago and reaching its present state around 300,000 years ago) separated high elevation habitat of both mountain ranges [42,43]. Our dating analysis suggests that *Grylloblatta *populations from the White Mountains (*T_MRCA _*median estimate ~179,800, 95% CI 17,320-505,900) may have been isolated during this period of subsidence. These are the first data to suggest multiple refugia existed in the Sierra Nevada, and on both sides of the crest, during glacial phases.

### Evolutionary History of Alpine Endemics

*Grylloblatta *lineages, clades and subclades in California are characterized by broadly overlapping periods of diversification. Using unscaled relaxed clock methods, median estimates of *T_MRCA _*reveal pulses at multiple nested levels in the phylogeny. However there is inherent error in these estimates and a lineage through time plot does not show synchronous pulses of diversification. Instead, the lineage through time analysis shows a high early diversification rate, a pronounced decline, and a very recent recovery in *Grylloblatta *diversification. The calibrated molecular clock rate places diversification times within Sierra Nevada *Grylloblatta *lineages during the Pleistocene glacial stages. Although some median estimates are in accord with specific events in the glacial chronology in the Sierra Nevada, confidence intervals are broad and overlap glacial-interglacial cycles. For example, median age estimates of within-population variation in alpine populations is ~150,000 years B.P., during the glacial maximum of the Illinoian advance, which was followed by an interglacial warm phase [23], but confidence intervals include the most recent glacial maximum (~25,000 years B.P.). Hence, the inherent uncertainty in the timing of diversification does not provide clear resolution to whether grylloblattid populations diversify during glacial advances or, alternatively, diversify during the retreat of alpine glaciers. Population genetic statistical tests provide equivocal support for recent population expansion in several well-sampled alpine *Grylloblatta *populations, although this may be an artifact of limited sample sizes. Furthermore, glacial phases in the Sierra Nevada are known to involve multiple glacial advances and retreats [44], which caused dynamic shifts in vegetation over short periods of time [23,45]. The pace and scale of such changes may not have favored periods of stable growth in *Grylloblatta *populations.

An older period of lineage diversification, corresponding to the *T_MRCA _*of recognized morphological species, dates to ~700,000-1,000,000 years B.P., well within the Middle Pleistocene epoch. Morphological criteria for distinguishing these species involve differences in male and female reproductive morphology, as well as differences in sensory structures and body size attributes. Changes in reproductive morphology are known to impede reproductive compatibility in insects [46], which suggests that multiple alpine *Grylloblatta *lineages have undergone recent speciation. The *T_MRCA _*of all California and all Sierra Nevada populations (~3.9-4.5 MYA) predates the Pleistocene. This suggests that Grylloblatta species may have colonized California during glacial advances of Pliocene epoch. It is likely that climatic oscillations led to the formation of discrete genetic populations, morphologically distinguishable taxa [47,48], and the first dispersal event into the Sierra Nevada during the evolution of California *Grylloblatta*.

## Conclusions

Recent speciation and the formation of multiple endemic lineages may be a common feature of alpine landscapes. This has been shown repeatedly in alpine taxa, as demonstrated in examples of North American grasshoppers [49], New Zealand plants and insects [50], and European plants [51]. These studies provide evidence that Pleistocene climate change has acted as a species pump [52] in alpine environments. California *Grylloblatta *provide an example of lineage diversification during glacial expansion events on a very small geographical scale. This highlights the importance of multiple refugia in mountains as sources of contemporary genetic variation in alpine species.

## Methods

### Genetic sampling

Sampling was done between 2005 and 2008 throughout California, with a particular focus on alpine populations in the Sierra Nevada. Despite systematic efforts to sample *Grylloblatta *[[34,53]; Schoville, S.D. unpublished data, [54]], populations are highly fragmented and local population densities appear to be low. Genetic samples comprise thirty sites and a total of 134 individuals (Figure 1; Additional file 1 Table 1S; Additional file 2 Table 2S). The majority of populations sampled are new locality records, including most Sierra Nevada collecting sites. Relevant genetic samples were taken from Jarvis and Whiting [39], including four California sites and two sites from Oregon. An individual of *Grylloblatta *from Oregon Cave, Oregon is used as an out-group based on the phylogenetic results of Jarvis and Whiting [39]. When possible, individuals were assigned to species based on previous phylogenetic hypotheses [34,39] and using morphological criteria [47,48,55], but populations from new sites within the Sierra Nevada have not been assigned to previously described species. All specimens have been deposited in the Essig Museum of Entomology, University of California, Berkeley.

### Molecular techniques and data preparation

Genomic DNA was extracted from 1-2 legs of each individual with a Qiagen DNEasy Blood and Tissue Kit (Qiagen) with reduced elution volumes. To maintain consistency with previously collected datasets [39], genetic data were collected from one mitochondrial and three nuclear genes. A region of approximately 790 bp representing the entire mitochondrial *cytochrome oxidase subunit II *(*COII*) gene was amplified and sequenced in both directions with the primers COII-F-Leucine and COII-R-Lysine [56]. Three nuclear genes were also amplified, including *histone *(*H3*) and the *18 S *and *28 S *ribosomal genes. A 380 bp region of the *histone *gene was amplified and sequenced in both directions using the primers HexA-F and HexA-R [56]. 2,100 bp of *18 S *and 2,450 bp of *28 S *were amplified using several overlapping primer combinations [57]. PCR products were sequenced in both directions on an ABI 3730 capillary sequencer using Big Dye v3.1 chemistry (Applied Biosystems).

DNA sequences were manually edited using the program SEQUENCHER v4.8 (Gene Codes Corporation). Some individuals were heterozygous at *histone *(*H3*) and haplotypes phase was estimated using the program PHASE v2 [58]. Visual alignments were made for each gene in the program MACLADE v4.08 [59]. Gaps were evident in both 18 S and 28 S and these were retained in subsequent phylogenetic analysis.

### Population Genetic Analysis of Alpine Populations

Changes in demographic history are known to affect the frequency of alleles, the distribution of mutations, and the coalescent times of gene copies. Population summary statistics examine genetic variability within populations and can be used to test for departures from demographic equilibrium. Statistical tests using Tajima's *D*, Fu's *F_S_*, and the mismatch distribution *SSD *and Raggedness Index were calculated in ARLEQUIN v3.11 [60]. The *D *and *F_S _*neutrality tests are sensitive to changes in demography or selection [61,62]. Significant negative values indicate population growth or purifying expansion, while significant positive values indicate population bottlenecks or balancing selection. Mismatch distributions model the frequency distribution of pairwise differences in a sudden population expansion [63], as well as under a spatial population expansion model [64,65]. Significant *SSD *or Raggedness Index values indicate that an expansion model is rejected by the data. Although most of the *Grylloblatta *sample sites have too few individuals to calculate population genetic summary statistics, five alpine localities contain sample sizes of greater than ten individuals (Barker Pass, Susie Lake, Sonora Pass, White Mountains and Graveyard Lakes). Each of the summary statistics was calculated for *COII *and *histone *in these five localities. Significance values for all four statistical tests were calculated using 1,000 bootstrap replicates.

### Phylogenetic Analysis

Phylogenetic relationships of California *Grylloblatta *were estimated using an unrooted Bayesian method and a maximum likelihood bootstrapping method. The four genes were concatenated and partitioned in each analysis. *COII *was further partitioned by codon position. MRMODELTEST2 v2.3 [66] was used to estimate models of molecular evolution for each partition. Based on Akaike Information Criteria [AIC, [67]], the best models for each partition were: *COII_pos1 _*GTR+G+I, *COII_pos2 _*GTR+I, *COII_pos3 _*GTR+G+I, *histone *HKY+I, *18 S *GTR+I, and *28 S *GTR+I. *Histone *was not partitioned by codon due to the lack of variable sites at first and second codon positions.

The program MRBAYES v3.1.2 [68] was used to estimate a Bayesian phylogeny of the concatenated and partitioned dataset (n = 87). Polymorphic sites at nuclear genes were treated as base ambiguities. Two independent analyses were run under the following conditions: 15 million steps, four chains, and genealogies sampled every 1000 steps. Performance of the Monte Carlo Markov Chain simulations was assessed for convergence using AWTY [69]. The first 4,000 samples were discarded as burn-in samples and a 50% majority rule consensus tree of the two independent runs was produced with posterior probability values equal to bipartition frequencies. A maximum-likelihood tree was also estimated using a rapid bootstrap heuristic in RAxML v7.0.4 [70]. A single run was conducted with 1000 bootstrap replicates followed by a full maximum likelihood search, using the partitioned data with the GTR+G+I model selected for each partition and the Oregon Cave sample selected as an out-group. Additionally, an mtDNA gene tree and a concatenated nuclear gene tree were estimated using MRBAYES with the following conditions: data partitioned by codon, two separate runs of 20 million steps each, four chains, and genealogies sampled every 1000 steps. The first 5,000 samples were discarded as burn-in samples and a 50% majority rule consensus tree of the two runs was produced with posterior probability values equal to bipartition frequencies.

The concatenated phylogeny was plotted on a digital elevation base map of North American using GENGIS v.1.05 [71].

### Timing of Lineage Diversification

Coalescent theory can be used to estimate the age of a monophyletic lineage by calculating the time to most recent common ancestor (*T_MRCA_*). This approach was used to calculate the age of the *Grylloblatta *lineages, as well as the time to a most recent common ancestor of sister clades. The estimation of absolute ages requires translation of molecular substitution rates into average rates per year. These rates cannot currently be estimated using fossil calibration because no there are no known fossils within the extant Grylloblattidae or of recent ancestral stem groups [72]. Mitochondrial substitution rates are available from fossil-based studies of other insect lineages [73,74]. These studies have shown a convergence in *cytochrome oxidase subunit I *mutation rates at 1.5% per million years. Molecular substitution rates are similar between *cytochrome oxidase subunit I *and *cytochrome oxidase subunit II *in insects, and the same rate is often applied to both genes [e.g [75,76]].

In order to explicitly address the uncertainty in using imported substitution rates, a relaxed clock model was selected for a Bayesian phylogenetic analysis in BEAST v1.5.3 [77,78]. Under this model, substitution rates are allowed to vary within a lineage and across lineages within the dataset under the constraints of a prior distribution. Two alternative priors were chosen to explore the divergence time of California *Grylloblatta*. First, the mean rate of evolution across the whole tree fixed to a mean of 1 in order to provide unscaled *T_MRCA _*estimates [78]. Second, a mean substitution rate estimate of 1.5% per million years was applied to *COII*. Uncertainty was added to the substitution rate by setting the standard deviation to 0.5% (mean = 0.015, 95% interval range: 0.00678 to 0.0232 per million years). The multi-locus dataset was partitioned by gene using the following substitution models: *COII *GTR+G+I, *histone *HKY+I, *18 S *GTR+I, and *28 S *GTR+I. The relaxed clock model with an uncorrelated log-normal distribution was chosen as a prior for rate variation among lineages and the Yule speciation process was chosen as a tree prior. Independent analyses were conducted under the following conditions: 100 million steps, genealogies sampled every 1000 steps, and a burn-in period of 10 million steps. Median estimates of time to most recent common ancestor (*T_MRCA_*) and 95% confidence intervals were calculated within the phylogeny using TRACER v1.5.

Lineage through time plots were constructed using the unscaled ultrametric phylogeny from the relaxed clock analysis. The library APE [79] in the software R v2.10.1 [80] was used to plot log-transformed lineage diversification versus time, relative to null model with a constant rate of speciation.

## Authors' contributions

SDS conducted the field work, laboratory work, genetic analysis and wrote the manuscript. GKR provided critical comments on the analysis and drafting of the manuscript. Both authors read and approved the final manuscript.

## Authors' Information

Sean Schoville's research focuses on population history, climate change and conservation of alpine insects. This work was completed as part of his PhD research on alpine biogeography. George Roderick is a Professor in the Department of Environmental Science, Policy and Management. His research interests include historical population dynamics, invasion biology, and biodiversity, mostly of insects.

## Supplementary Material

Additional file 1 **Table 1S.** Collecting localities of *Grylloblatta*. Latitude and longitude reported in the WGS84 datum. Type localities indicated with an asterisk. Click here for file

Additional file 2 **Table 2S.** GenBank Accession numbers of California *Grylloblatta *collections. Click here for file

Additional file 3 **Figure 1S.** Mitochondrial gene tree of California *Grylloblatta *species. Bayesian posterior probability support values are shown at the base of the nodes. Click here for file

Additional file 4 **Figure 2S.** Concatenated nuclear gene tree of California *Grylloblatta *species. Bayesian posterior probability support values are shown at the base of the nodes. Click here for file

Additional file 5 **Figure 3S.** Lineage through time plot of California *Grylloblatta *species. Plot of log-lineage diversification (solid line) over unscaled time, compared to a null model (dashed line) of constant diversification. Click here for file
