# Supplementary material for: Evolutionary diversification of cryophilic Grylloblatta species (Grylloblattodea: Grylloblattidae) in alpine habitats of California
Source: BMC Evol Biol. 2010 Jun 2;10:163. doi: 10.1186/1471-2148-10-163 (PMC2898686; doi:10.1186/1471-2148-10-163)
Supplement: Additional file 1 — Table 1S. Collecting localities of Grylloblatta. Latitude and longitude reported in the WGS84 datum. Type localities indicated with an asterisk. [file 1471-2148-10-163-S1.PDF]

Additional Table 1S. Collecting localities of *Grylloblatta* .

Latitude and longitiude reported in the WGS84 datum. Type localities indicated with an asterisk.

Site #	Species/Clade	Locality	Latitude	Longitude
1	*G. rothi*	McKenzie Pass	44.26	-121.809
2	Oregon Cave	Oregon Cave	42.098	-123.406
3a	*G. gurneyi*	Merrill Ice Cave	41.728	-121.547
3b	*G. gurneyi*	Cox Cave	41.715	-121.498
4	*G. chandleri*	Gray's Flat	40.628	-121.18932
5	*G. chandleri*	Wilson Ice Cave	40.33592	-121.42169
6	*G. barberi*	Caribou Powerhouse*	40.09134	-121.13687
7	*G. washoa*	Carpenter Ridge	39.4126	-120.32047
8	*G. washoa*	SE Barker Pass	39.06661	-120.22999
9	*G. washoa*	Susie Lake	38.88574	-120.1345
10	*G. washoa*	Smith Lake	38.85681	-120.18805
11	*G. bifratrilecta*	Carson Pass	38.66666	-119.99001
12	*G. bifratrilecta*	Sonora Pass*	38.31556	-119.66673
13	Tioga Crest	Greenstone Lake	37.97636	-119.29442
14	Tioga Crest	Granite Lakes	37.92809	-119.28494
15	Tioga Crest	Vogelsang Lake	37.78619	-119.34689
16	Tioga Crest	Mt. Lyell	37.76657	-119.25471
17	Ostrander Lake	Ostrander Lake	37.63543	-119.58054
18	Ostrander Lake	Indian Cave	37.748	-119.549
19	Central Sierra Nevada	White Mountains	37.62884	-118.24305
20	Central Sierra Nevada	Lake George	37.59764	-119.01425
21a	Central Sierra Nevada	Graveyard Lakes	37.44576	-118.97408
21b	Graveyard Lakes	Graveyard Lakes	37.44576	-118.97408
22	Central Sierra Nevada	Harvey Lake	37.30436	-118.91287
23	Central Sierra Nevada	Selden Pass	37.28555	-118.88175
24	Central Sierra Nevada	Sam Mack Lake	37.11646	-118.51212
25	Central Sierra Nevada	Sixty Lake Basin	36.82479	-118.43382
26	Central Sierra Nevada	Sphinx Lakes	36.71427	-118.51487
27	Lillburn Cave	Lillburn Cave	36.655	-118.907
28	Southwest Sierra Nevada	Mt. Silliman	36.63881	-118.69416
29	Southwest Sierra Nevada	Pear Lake	36.59755	-118.66609
30	Southwest Sierra Nevada	Monarch Lakes	36.44792	-118.55756
