# Supplementary material for: Evolutionary diversification of cryophilic Grylloblatta species (Grylloblattodea: Grylloblattidae) in alpine habitats of California
Source: BMC Evol Biol. 2010 Jun 2;10:163. doi: 10.1186/1471-2148-10-163 (PMC2898686; doi:10.1186/1471-2148-10-163)
Supplement: Additional file 2 — Table 2S. GenBank Accession numbers of California Grylloblatta collections. [file 1471-2148-10-163-S2.PDF]

Additional Table 2S. GenBank Accession numbers of California *Grylloblatta* collections.

		Cytochrome oxidase subunit II		Histone 3		28S ribosomal RNA		18S ribosomal RNA	
Site #	N	Accession #	Frequency	Accession #	Frequency	Accession #	Frequency	Accession #	Frequency
1	1	DQ457341	1	DQ457371	1	DQ457306	1	DQ457269	1
2	1	DQ457366	1	DQ457397	1	DQ457335	1	DQ457298	1
3a	1	DQ457347	1	DQ457378	1	DQ457313	1	DQ457276	1
3b	1	DQ457349	1	DQ457380	1	DQ457315	1	DQ457278	1
4	1	FJ918575	1	FJ918628	1	FJ918685	1	FJ918665	1
5	1	DQ457340	1	DQ457370	1	DQ457305	1	DQ457268	1
6	1	FJ918624	1	FJ918645	1	FJ918686	1	FJ918664	1
7	1	not available	0	DQ457390	1	DQ457325	1	DQ457288	1
8	17	FJ918620<br>FJ918621<br>FJ918622<br>FJ918623	0.53<br>0.06<br>0.35<br>0.06	FJ918633<br>FJ918639	0.82<br>0.18	FJ918667<br>FJ918668	0.97<br>0.03	FJ918648	1
9	21	FJ918607<br>FJ918608<br>FJ918609<br>FJ918610<br>FJ918611<br>FJ918612<br>FJ918613<br>FJ918614<br>FJ918615<br>FJ918616	0.14<br>0.38<br>0.1<br>0.05<br>0.05<br>0.05<br>0.05<br>0.05<br>0.05<br>0.05	FJ918633<br>FJ918639<br>FJ918644	0.96<br>0.02<br>0.02	FJ918667<br>FJ918668	0.98<br>0.02	FJ918648<br>FJ918659<br>FJ918660	0.96<br>0.02<br>0.02
10	2	FJ918578<br>FJ918579	0.5<br>0.5	FJ918633	1	FJ918667<br>FJ918668	0.75<br>0.25	FJ918648	1
11	7	FJ918581<br>FJ918617<br>FJ918618	0.71<br>0.14<br>0.14	FJ918626	1	FJ918666<br>FJ918681	0.71<br>0.29	FJ918647<br>FJ918661	0.78<br>0.21
12	12	FJ918584<br>FJ918585<br>FJ918586<br>FJ918587	0.42<br>0.33<br>0.17<br>0.08	FJ918626	1	FJ918671<br>FJ918672	0.33<br>0.67	FJ918661	1
13	9	FJ918580<br>FJ918625	0.89<br>0.11	FJ918632	1	FJ918680<br>FJ918682<br>FJ918683<br>FJ918684	0.39<br>0.11<br>0.44<br>0.06	FJ918649<br>FJ918662<br>FJ918663	0.84<br>0.06<br>0.11
14	1	FJ918580	1	FJ918635	1	FJ918669	1	FJ918649	1
15	1	FJ918582	1	FJ918635	1	FJ918669	1	FJ918650	1
16	1	FJ918605	1	FJ918632	1	FJ918680	1	FJ918649	1
17	1	FJ918583	1	FJ918630	1	FJ918670	1	FJ918651	1
18	1	GU013769	1	GU013770	1	FJ918670	1	FJ918651	1
19	11	FJ918588<br>FJ918589	0.91<br>0.09	FJ918627<br>FJ918636	0.91<br>0.09	FJ918673<br>FJ918675	0.82<br>0.18	FJ918653	1
20	1	FJ918619	1	FJ918627<br>FJ918642	0.5<br>0.5	FJ918673	1	FJ918653	1
21a	1	FJ918591	1	FJ918640	1	FJ918678	1	FJ918657	1
21b	12	FJ918592<br>FJ918593<br>FJ918599	0.67<br>0.25<br>0.8	FJ918627<br>FJ918636<br>FJ918641<br>FJ918642	0.42<br>0.04<br>0.5<br>0.04	FJ918673	1	FJ918653	1
22	1	FJ918595	1	FJ918634<br>FJ918643	0.5<br>0.5	FJ918673	1	FJ918655	1

23	3	FJ918600<br>FJ918601	0.33<br>0.67		FJ918627<br>FJ918638<br>FJ918642	0.5<br>0.33<br>0.17		FJ918673<br>FJ918675	0.83<br>0.17		FJ918653	1
24	1	FJ918598	1		FJ918637<br>FJ918638	0.5<br>0.5		FJ918679	1		FJ918655	1
25	6	FJ918590	1		FJ918627<br>FJ918636<br>FJ918637<br>FJ918638	0.17<br>0.25<br>0.5<br>0.08		FJ918673	1		FJ918655	1
26	7	FJ918590<br>FJ918602	0.86<br>0.14		FJ918634<br>FJ918636<br>FJ918642	0.07<br>0.43<br>0.5		FJ918673	1		FJ918655	1
27	1	FJ918576<br>FJ918577	**		FJ918629	1		FJ918687	1		FJ918652	1
28	6	FJ918595<br>FJ918596	0.83<br>0.17		FJ918631<br>FJ918643	0.75<br>0.25		FJ918674	1		FJ918654<br>FJ918656<br>FJ918658	0.08<br>0.67<br>0.25
29	5	FJ918595<br>FJ918597<br>FJ918603	0.6<br>0.2<br>0.2		FJ918631<br>FJ918643	0.7<br>0.3		FJ918674	1		FJ918654<br>FJ918656<br>FJ918658	0.2<br>0.6<br>0.2
30	1	FJ918604	1		FJ918631	1		FJ918677	1		FJ918654	1
