# Supplementary material for: Evolutionary diversification of cryophilic Grylloblatta species (Grylloblattodea: Grylloblattidae) in alpine habitats of California
Source: BMC Evol Biol. 2010 Jun 2;10:163. doi: 10.1186/1471-2148-10-163 (PMC2898686; doi:10.1186/1471-2148-10-163)

Additional Figure 2S. Nuclear concatenated tree of California *Grylloblatta* species.
Bayesian posterior probability support values are shown at the base of the nodes.

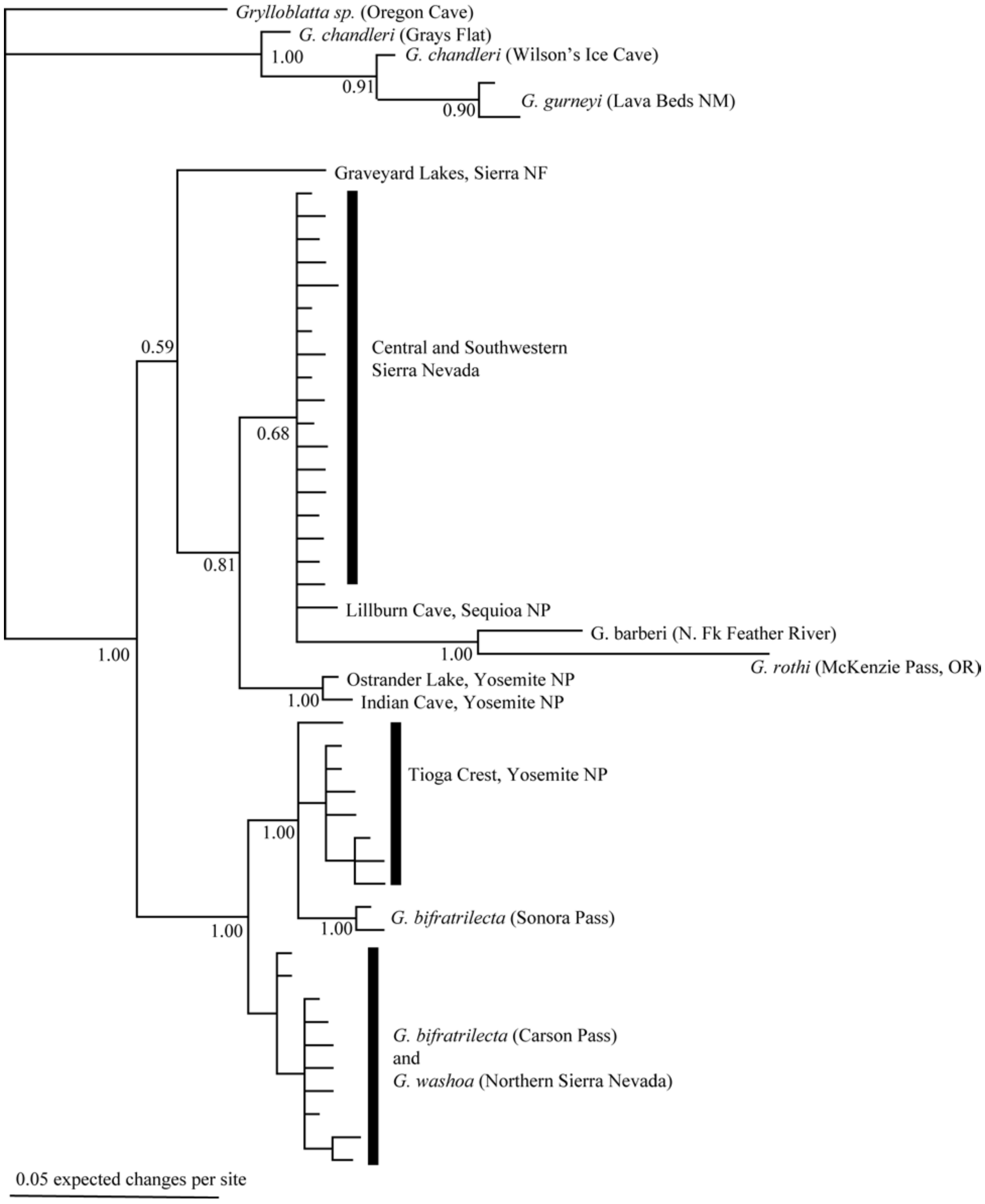

Supplement: Additional file 4 — Figure 2S. Concatenated nuclear gene tree of California Grylloblatta species. Bayesian posterior probability support values are shown at the base of the nodes. [file 1471-2148-10-163-S4.PDF]
