# Supplementary material for: Evolutionary diversification of cryophilic Grylloblatta species (Grylloblattodea: Grylloblattidae) in alpine habitats of California
Source: BMC Evol Biol. 2010 Jun 2;10:163. doi: 10.1186/1471-2148-10-163 (PMC2898686; doi:10.1186/1471-2148-10-163)

**Additional Figure 3S. Lineage through time plot of California *Grylloblatta* species.**
Plot of log-lineage diversification (solid line) over unscaled time, compared to a null model (dashed line) of constant diversification.

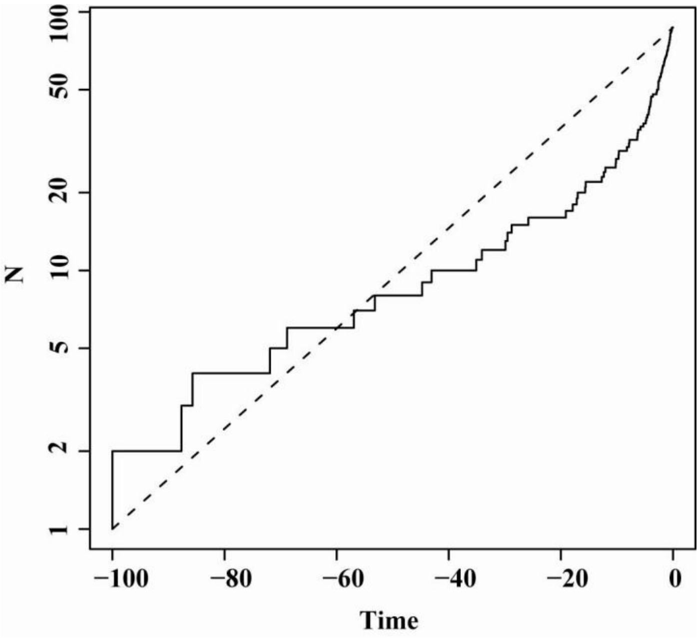

Supplement: Additional file 5 — Figure 3S. Lineage through time plot of California Grylloblatta species. Plot of log-lineage diversification (solid line) over unscaled time, compared to a null model (dashed line) of constant diversification. [file 1471-2148-10-163-S5.PDF]
